# Offshore aquaculture: Spatial planning principles for sustainable development

**DOI:** 10.1002/ece3.2637

**Published:** 2016-12-24

**Authors:** Rebecca R. Gentry, Sarah E. Lester, Carrie V. Kappel, Crow White, Tom W. Bell, Joel Stevens, Steven D. Gaines

**Affiliations:** ^1^ Bren School of Environmental Science & ManagementUniversity of California Santa BarbaraSanta BarbaraCAUSA; ^2^ Department of GeographyFlorida State UniversityTallahasseeFLUSA; ^3^ National Center for Ecological Analysis and SynthesisSanta BarbaraCAUSA; ^4^ Center for Coastal Marine SciencesCalifornia Polytechnic Institute San Luis ObispoSan Luis ObispoCAUSA; ^5^ Earth Research InstituteUniversity of California Santa BarbaraSanta BarbaraCAUSA

**Keywords:** cumulative impacts, disease, environmental effects, fisheries, mariculture, marine spatial planning, ocean zoning, open‐ocean aquaculture, tradeoffs

## Abstract

Marine aquaculture is expanding into deeper offshore environments in response to growing consumer demand for seafood, improved technology, and limited potential to increase wild fisheries catches. Sustainable development of aquaculture will require quantification and minimization of its impacts on other ocean‐based activities and the environment through scientifically informed spatial planning. However, the scientific literature currently provides limited direct guidance for such planning. Here, we employ an ecological lens and synthesize a broad multidisciplinary literature to provide insight into the interactions between offshore aquaculture and the surrounding environment across a spectrum of spatial scales. While important information gaps remain, we find that there is sufficient research for informed decisions about the effects of aquaculture siting to achieve a sustainable offshore aquaculture industry that complements other uses of the marine environment.

## 1 Introduction

Aquaculture is currently the fastest growing food sector in the world, and the open oceans are seen as one of the most likely areas for large‐scale expansion (Lovatelli, Aguilar‐Manjarrez, & Soto, 2013; Rubino, 2008). The global demand for seafood is continuing to rise sharply, driven by both population growth and increased per capita consumption (Godfray et al., 2010). Wild‐capture fisheries are constrained in their potential to produce more seafood (Costello et al., 2016) making aquaculture growth the most likely scenario to meet the majority of increased demand (Goldburg & Naylor, 2005).

Traditionally, mariculture has taken place at the land–sea interface—in intertidal areas, estuaries, and sheltered bays. While calm waters and easy access make nearshore seafood farming attractive, some environmental impacts and conflicts with other uses are accentuated in the increasingly crowded coastal zone. Advances in technology and culture methods have made it possible to establish farms further from shore and in rougher open‐ocean conditions, opening up new expanses to potential aquaculture farming (Bostock et al., 2010; Shainee, Haskins, Ellingsen, & Leira, 2012). Offshore aquaculture offers promise for increasing the supply of seafood and as a source of new economic development.

Ensuring sustainable management of this emerging industry requires an understanding of how marine aquaculture, or ‘mariculture,’ interacts with the surrounding environment and how the location and density of development affects both aquaculture value and the health and productivity of the surrounding ecosystem. Mariculture development has raised many environmental concerns, including habitat destruction (Ottinger, Clauss, & Kuenzer, 2016), pollution (Islam, 2005), introduction of disease (Lafferty et al., 2015), interbreeding of escapees with wild stocks (Naylor, Williams, & Strong, 2001), entanglement of marine mega‐fauna (Kemper et al., 2003), and the sustainability of fish‐derived feeds (Naylor et al., 2009); many of these impacts have been well studied across a variety of cultures and environments. Although farm practices (e.g., low stocking density, reduced feed waste, preventative veterinary care) can play a major role in ensuring good environmental outcomes (Cho & Bureau, 2001; Wu, 1995), the choice of farm location also plays a critical role in determining its productivity, environmental impact, and interactions with other ecosystem services provided by the ocean.

Scientists and policymakers have recommended spatial planning as an approach to comprehensively consider multiple uses and values of the marine environment (Calado et al., 2010; Lester et al., 2013; Obama, 2010). Although ocean planning lags behind terrestrial planning, the spatial complexity and dynamics of the ocean environment make spatial planning particularly important (Crowder & Norse, 2008). Most siting for aquaculture, like other uses of marine space, has been undertaken on an ad hoc basis for a single farm or collection of farms without integrated or broader strategic planning (Douvere, 2008), and many “comprehensive” spatial planning processes fail to explicitly plan for offshore aquaculture. However, there is an increasing emphasis on the need for proactive planning and zoning for mariculture in locations across the globe (Aguilar‐Manjarrez, Kapetsky, & Soto, 2010). A growing number of national and regional authorities are beginning to engage in aquaculture planning processes or wider marine spatial planning processes that involve aquaculture (Sanchez‐Jerez et al., 2016), highlighting the need for more comprehensive scientific guidance.

Proactive spatial planning is essential for successful and sustainable mariculture development because many of the interactions between aquaculture farms and the surrounding ecosystem vary significantly with location. These interactions can have strong impacts on both the mariculture operation and on other uses and values in the marine environment; in some instances, ecosystem effects of mariculture can be seen far beyond the footprint of the farm. Although there are many important aspects of aquaculture sustainability related to supply chains and farm practices, here we focus on spatial planning considerations for aquaculture development. We outline ways in which offshore aquaculture interacts with the surrounding environment and assess which aspects of offshore aquaculture sustainability are important from a spatial planning perspective, at both the scales of individual site selection and regional planning. Finally, we suggest relevant tools and planning approaches for guiding sustainable offshore aquaculture siting.

Although we highlight gaps in current knowledge, our primary goal is to demonstrate the substantial body of knowledge, from across disciplines, that informs our understanding of aquaculture interactions with the surrounding environment and how this understanding can be used to inform spatial planning. This includes assessment of tools that have primarily been used for aquaculture in shallow sheltered environments and their relevance for more open‐ocean conditions. By synthesizing this knowledge, we are able to clarify key risks and opportunities related to aquaculture planning, even when data are limited. We suggest that the location of marine aquaculture development has a significant impact on its potential environmental effects and suitability within a region, and thus, spatial planning can make a large difference in creating positive outcomes. We add to the growing literature on ecosystem‐based management of our oceans and create a platform for considering the role of sustainable aquaculture development as a part of healthy and productive seascapes.

## 2 Spatial Considerations for Offshore Aquaculture Development

**Figure 1:**
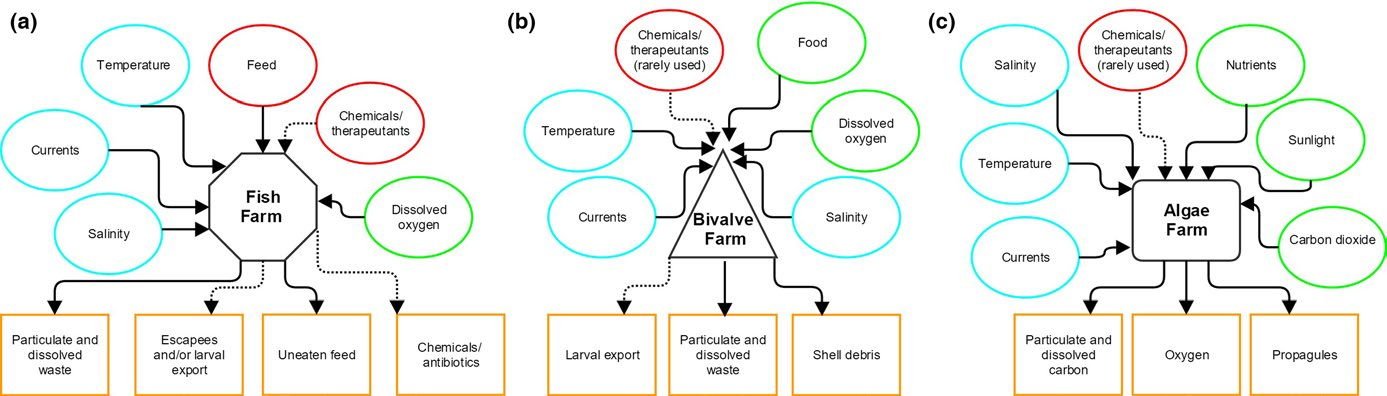
Schematic of key inputs and outputs associated with the three major categories of aquaculture: (a) fed, (b) unfed, and (c) autotrophic. Red indicates external inputs into the farm; green indicates environmental inputs; blue indicates other environmental conditions that affect the farm; and orange indicates outputs from the farm into the environment. Dashed lines indicate inputs and outputs that are only sometimes present

Offshore aquaculture has been defined using a variety of criteria, including water depth, distance from shore, wave exposure, and jurisdictional boundaries (Holmer, 2010; Kapetsky, 2013; Rubino, 2008); here, we use a broad definition that includes all mariculture that is located in open water (i.e., not directly adjacent to land or within a bay or fjord). There is significant diversity in marine aquaculture species, with nearly 200 species currently being farmed (FAO 2015) and many more under development; however, all types of mariculture fall into three broad categories: fed (e.g., fish, most crustaceans), unfed (e.g., filter‐feeding bivalves, some grazers, and detritivores), and autotrophic species (kelp and other algae). Each of these culture categories interacts with the environment in fundamentally different ways, both in terms of external inputs to the farm and effects of the farm on its surrounding environment (Figure 1). As aquaculture moves into new frontiers—both geographically and technologically—there is an important opportunity to determine where to pursue offshore development in the context of the ocean's complex ecological dynamics and the diversity of existing marine activities and benefits that could interact with or be impacted by aquaculture. We examine four categories of spatial interactions between offshore aquaculture, the environment, and other uses: effects of the environment on farms; effects of farms on the environment; cumulative impacts and regional planning issues; and synergies and conflicts with other ocean management goals.

### 2.1 Effects of the environment on farms

An essential consideration for offshore aquaculture planning is determining which areas could be most productive and profitable. The suitability of locations varies widely, even over small distances. Physical factors, such as water temperature, ocean currents, sunlight, and food and nutrient availability, have a direct effect on the growth of aquaculture species (Ferreira, Hawkins, & Bricker, 2007). Unfed and autotrophic aquaculture species are particularly sensitive to environmental conditions because they rely on the surrounding environment to provide the energy needed for growth. Available oceanographic data can be integrated into species‐specific growth functions to compare the suitability of potential sites for maximizing growth. There are also several software applications that can model site‐level production for specific aquaculture species, such as the FARM model (Ferreira et al., 2007), ShellSim (Hawkins et al., 2013), Depomod (Cromey, Nickell, & Black, 2002), and Aquamodel (Rensel, Kiefer, Forster, Woodruff, & Evans, 2007). While these models are designed for modeling site‐specific production and impact, they can also be utilized to determine the areas of highest production within a region by running the model across a spectrum of sites. This type of spatial comparison of productivity has been applied to nearshore bivalve aquaculture in Chile and Scotland (Ferreira et al., 2008; Silva et al., 2011) and to offshore aquaculture in the Southern California Bight (S. Lester, personal communication, 2016). Generally, this type of approach requires significant environmental and farm level data, such as currents, primary productivity, temperature, and stocking density, which can limit its broad application in areas with limited environmental information.

Farm location also impacts the quality of seafood produced. Notably, concerns about the accumulation of toxins in seafood are driving efforts to ensure the safety of aquaculture products (Focardi, Corsi, & Franchi, 2005; Karunasagar, 2008). Existing research on the distribution and impacts of land‐based pollutants on marine ecosystems (e.g., Fabricius, 2005; Halpern et al., 2009) and monitoring of water quality could help inform offshore aquaculture planning. For example, Fabricius (2007) detail spatial, physical, and hydrodynamic properties of the environment that are likely to affect the susceptibility of coral reefs to the effects of land‐based runoff. Many of the characteristics of susceptible reef areas, such as close proximity to discharge, shallow depths, and slow currents, are also likely to be risk factors for aquaculture operations. In general, moving into offshore environments, which is likely to increase the distance from most pollution sources and to increase water flow, will be beneficial in mitigating food safety concerns. Evidence from bluefin tuna ranching in Australia suggests that moving marine aquaculture into offshore environments may also enhance fish condition, while reducing parasite loads and mortality rates (Kirchhoff, Rough, & Nowak, 2011).

Farm productivity and profit can also be impacted by wild predators, such as seals, sea lions, otters, and birds, that are often attracted to mariculture farms. For example, predator presence near farms can generate stress‐related fitness reductions in farmed fish, damage to farms, and increased escapement of farmed fish from damaged nets (Nash, Iwamoto, & Mahnken, 2000). These interactions can be minimized through cage design and auditory or other deterrents (Quick, Middlemas, & Armstrong, 2004), but location of the farm is also important. For example, evidence from both Australia and Chile suggests that predation rates on an aquaculture farm are related to distance from the nearest pinniped colony (Kemper et al., 2003). In general, moving farms further offshore and away from coastal concentrations of marine mammals is likely to help minimize interactions and protect the cultured product from predation (Nash et al., 2000).

Farm location can also have a significant impact on the cost of farm operations. Factors such as depth, distance from port (and associated infrastructure and processing facilities), wave conditions, and storm activity modify transport, labor, construction, and maintenance costs (Kaiser, Snyder, & Yu, 2011; Klinger & Naylor, 2012). Additionally, risks due to climate variability, pollution, disease, and harmful algal blooms can vary spatially (e.g., Husson, Hernández‐Fariñas, Le Gendre, Schapira, & Chapelle, 2016) and may have an effect on the profitability of a farm.

### 2.2 Effects of farms on the environment

By introducing a high density of additional life into the ocean, mariculture affects the surrounding environment in diverse and complex ways. In some cases, this can lead to desirable outcomes; for example, algal aquaculture has the potential to improve water quality in regions that have been affected by nutrient pollution through uptake of nitrogen, phosphorous, and carbon (Neori et al., 2004). Bivalves have also been promoted for their ability to reduce the standing stock of phytoplankton, and therefore potentially mitigate some of the effects of eutrophication (Cranford, Dowd, & Grant, 2003). However, aquaculture can also contribute to nutrient and chemical pollution (Cao et al., 2007). The magnitude of these effects is heavily influenced by operational characteristics, such as the species farmed, stocking density, and feeding strategy, but location also plays an important role. Specifically, physical and chemical characteristics of the surrounding environment, such as background nutrient levels, proximity to sensitive habitats, currents, and depth, help to determine the fate and impact of pollutants released from a farm.

Both fed and unfed aquaculture operations can release particulate organic matter that is likely to fall to the seafloor, potentially leading to local oxygen depletion in and near the benthos as the organic matter is consumed by microbes (Ferreira et al., 2007; Price & Morris, 2013). Generally, deeper water and faster currents result in more diffusion of organic material (Lovatelli et al., 2013; Sarà, Scilipoti, Milazzo, & Modica, 2006). For example, a study examining ten aquaculture sites across Europe found that shallower depths and slower current speeds were significant predictors of higher levels of benthic impact; these hydrodynamic variables were second only to the amount and duration of aquaculture production in predictive strength (Borja et al., 2009). In general, while bivalve farms have been shown to have benthic impacts in shallow sheltered areas, there are low risks of significant organic enrichment in well‐managed marine farms, especially in areas of high current and depth (typical of offshore sites) (Crawford, 2003; Crawford, Macleod, & Mitchell, 2003). The potential benthic impacts of offshore finfish farming are less clear, and can vary significantly with farm practices (such as stocking density) and site characteristics (Price & Morris, 2013). While high levels of nutrient enrichment can cause adverse hypoxic conditions, low levels of nutrient enrichment may only have a minor effect and can actually result in an increase in benthic diversity (Rosenberg, Agrenius, Hellman, Nilsson, & Norling, 2002).

One possible approach to mitigate pollution from finfish farms is through integrated multitrophic aquaculture (IMTA), which aims to imitate natural ecological nutrient cycling by pairing different trophic levels of aquaculture in the same area (Neori et al., 2004; Troell et al., 2009). Fed aquaculture produces excess organic matter, which can feed bivalve aquaculture both directly and indirectly (i.e., by encouraging additional phytoplankton growth). In addition, fish and bivalves also produce dissolved nutrients that are necessary, and often limiting, for the growth of autotrophs. Therefore, placing unfed and autotrophic aquaculture in the same location as or adjacent to fed aquaculture could theoretically improve growing conditions for bivalves and kelp while mitigating some of the potential impacts of fed aquaculture. However, commercial operationalization of this idea in the offshore environment is relatively new and faces challenges with efficiency and economic scaling (Troell et al., 2009). The potential effectiveness of IMTA depends on environmental context, particularly background nutrient levels, food availability, and hydrodynamics (Troell et al., 2009).

Another environmental concern associated with offshore aquaculture is potential negative interactions with marine mammals, birds, and other wildlife. Wildlife can be attracted to aquaculture farms and then get caught in lines and nets (Kemper et al., 2003). However, the frequency of entanglement is typically quite low, and in general, the risk of entanglement in aquaculture gear is less than the risks associated with fishing gear (Young, 2015). Conversely, there is also concern that farms may displace whales and dolphins, which could impact their access to foraging grounds or impede movement. Evidence from Western Australia supports this concern by demonstrating that bottlenose dolphins avoid oyster farming areas (Watson‐Capps & Mann, 2005). Information about home ranges, movements, and behaviors of local marine mammals in response to aquaculture farming can help inform aquaculture development and provide better understanding of the risks to wildlife.

### 2.3 Cumulative impacts and regional planning issues

As the density of aquaculture within an area increases, additional regional‐scale considerations emerge regarding the number of farms that can be supported as part of a healthy ecosystem. These considerations are quite different and conceptually almost opposite for fed and unfed aquaculture: cumulative effects of adding additional organic matter to the ecosystem for fed aquaculture vs. cumulative effects of organic removals from the system for unfed aquaculture.

For offshore finfish farms, there is considerable uncertainty about how pollution impacts scale with the concentration of farms, and at what density and in what environments eutrophication is likely to become significant (Cao et al., 2007; Klinger & Naylor, 2012). Much of what we know about nutrient enrichment from mariculture comes from studies of farms in sheltered coastal locations (e.g., McKinnon et al., 2010; Niklitschek, Soto, Lafon, Molinet, & Toledo, 2013), where limited water flow can amplify pollution problems. Since offshore sites tend to be less susceptible to nutrient enrichment due to increased water flow and depth, offshore locations should sustainably support a higher density of production than sheltered nearshore locations, particularly if conservative stocking densities are used. Nonetheless, both the environmental context, in terms of background nutrient concentrations, other sources of organic influx, and the strength of currents, as well as farm management, particularly stocking density and feeding practices, are important in determining whether larger scale nutrient enrichment is likely to be a concern in any given area. If cumulative pollution is considered a risk, aquaculture‐specific modeling software, such as Aquamodel (Rensel et al., 2007), can provide further insight on the potential for cumulative nutrient pollution issues by modeling the effluent from several farms within a region.

With unfed, specifically bivalve, aquaculture there is a farm density at which the cultured species will consume so much food from the water column that ecosystem function will be impacted. Potential impacts include reduced wild recruitment due to over consumption of planktonic larvae and reduced food availability for wild populations (Gibbs, 2004). Several studies, including by Jiang and Gibbs (2005) in New Zealand and by Byron, Link, Costa‐Pierce, and Bengtson (2011) in Rhode Island, have used Ecopath, an ecosystem modeling software, to assess both the effect of existing bivalve culture on the ecosystem and determine sustainable limits to future production. While this type of study is data intensive, it is a powerful approach for considering ecosystem‐level effects and providing an assessment of carrying capacity. In general, food competition between wild and farmed species is more likely to be a concern in regions with low primary productivity (Gibbs, 2004; Grant et al., 2007), although those regions are also less likely to experience intense development of unfed aquaculture. In addition, the high water flow typical of open‐ocean farms makes significant issues with food competition unlikely, except at very high farm densities. Similarly, local nutrient depletion is potentially possible in areas of very‐high‐density kelp culture, but this has not generally been an issue in kelp‐growing regions (Kraan, 2013).

The risk of disease outbreak is also a prominent concern with aquaculture development, particularly in terms of cumulative impacts from multiple farms in a region (Holmer, 2010; Leung & Bates, 2013). Although site selection is often seen as secondary to management and husbandry practices in reducing disease outbreaks, the spatial distribution of aquaculture farms can play an important role in modifying this risk (Murray & Gubbins, 2016; Salama & Murray, 2011). The diversity of potential diseases and the constant emergence of new disease threats make spatial planning to reduce disease risk challenging (Lafferty et al., 2015). Each disease is specific in terms of its biology, how far it is likely to spread, and the specificity of its targeted host. Host specificity is particularly important in determining whether any disease outbreak is a serious environmental concern that has potential to spread to wild populations or is likely to remain within aquaculture farms (and is primarily an economic issue). Unfortunately, there are still significant unknowns concerning the biology and spread of many emerging diseases that could affect aquaculture species. However, even without disease‐specific information, spatial planning can reduce disease risk. For example, reducing the size and density of farms and increasing the distance between farms can mitigate the risk of disease spread; generally, larger farms spaced further apart pose less risk than multiple smaller farms clustered closely together (Salama & Murray, 2011). Infectious salmon anemia (ISA) is one disease that has received considerable research attention due to its history of impact on the aquaculture industry. Researchers in Chile and Norway have found that ISA spread among farms is more likely when farms are clustered closely together and recommend a separation distance of at least five kilometers between farms (Jarp & Karlsen, 1997; Mardones, Perez, & Carpenter, 2009). These simple guidelines are especially useful for diseases that are not shared with wild stocks and could be refined considerably with specific information about both the environment and the disease of concern.

Importantly, it is not precisely the geographic proximity of farms that matters for disease spread, but rather their connectivity—in other words, the likelihood that infectious agents from one farm reach another farm. In addition to physical distance, current speed, and direction also determine site connectivity. Oceanographic models, such as Regional Ocean Modeling Systems (ROMS) (e.g., Dong, Idica, & McWilliams, 2009), can be used to evaluate connectivity by modeling the release of particles at any one location and tracing the likelihood of transport to all other locations (Simons, Siegel, & Brown, 2013). Indeed, a recent study demonstrated that water contact via current flow had the strongest explanatory power in describing the dynamics of pancreas disease spread between salmon farms in Norway (Stene, Viljugrein, Yndestad, Tavornpanich, & Skjerve, 2014). This approach can be useful for forecasting the risks of disease spread (Groner et al., 2016) and informing spatial planning to minimize the connectivity between aquaculture locations. Spatial risk assessment for disease spread can be combined with other models to assess overall production and ecological carrying capacity for a region (Ferreira, Saurel, Lencart e Silva, Nunes, & Vazquez, 2014). This approach also has the advantage of using a systems perspective to demonstrate how the location and density of farm development affects both other farms and the surrounding environment across a spectrum of scales and sustainability metrics.

In addition to minimizing connectivity among farms, locating farms away from dense or vulnerable wild populations may reduce the risk of disease exchange between wild stocks and farmed animals (Holmer, 2010). Wild populations are well documented as the source of most aquaculture diseases (via water exchange, feed, or broodstock), and even diseases that do not affect wild hosts can be problematic if transferred to an aquaculture setting (Lafferty et al., 2015). However, it is the risk of disease export from aquaculture to the wild that has created the most concern and controversy from an ecological perspective (Johansen et al., 2011). This risk may be heightened when the farmed species is native or related to a native species (Gross, 1998). While diseases do pose potentially severe risks to wild populations, the role of aquaculture as a source of these diseases is controversial, and considerable uncertainty around the dynamics of disease spread from farms to wild stocks remains (Lafferty et al., 2015).

### 2.4 Synergies and conflicts with other ocean management goals

The location of offshore aquaculture facilities could have significant impacts, both positive and negative, on other ocean management considerations, including shipping, fishing, recreation, and conservation. This web of interactions suggests the need to plan for multiple objectives in concert. One planning approach is to avoid siting aquaculture in the most important areas for other ocean uses. However, simply avoiding areas that are already being used for another purpose will not necessarily lead to the best outcomes. Using theory adapted from economics, tradeoff analysis can provide guidance on how spatial planning can be used to minimize the inherent conflicts associated with multiple overlapping goals and arrive at a suite of solutions that maximize overall value (Lester et al., 2013).

Spatial tradeoffs between aquaculture, marine fisheries, and conservation are highly intertwined and present challenges and opportunities across a spectrum of spatial scales. For one, most aquaculture farms exclude other commercial activities, including fishing, effectively creating a refuge for some marine species. Literature on marine protected area network design has emphasized the importance of connectivity between reserves in ensuring conservation and management objectives (Gaines, Gaylord, & Largier, 2003; Gaines, White, Carr, & Palumbi, 2010). Therefore, if aquaculture farms are well connected to other farms or to a network of protected areas, they could help bolster conservation. However, aquaculture is a leading source of marine invasive species (Molnar, Gamboa, Revenga, & Spalding, 2008), and also potentially introduces risks of pollution and disease. Therefore, locating a farm so that it is highly connected to protected areas could introduce increased environmental risk. One key question is the relative rates of spread of these different biological and chemical entities. While more is known about the dispersal of larvae than the infection patterns of marine diseases, we do know that some larvae have the potential to disperse far longer in the open ocean (Kinlan, Gaines, & Lester, 2005) than many viruses (Suttle, Chen, Suttle, & Chen, 1992). This suggests their scales of dispersal may also be much larger and presents interesting spatial planning opportunities to minimize unwanted connectivity over smaller spatial scales, while maximizing desired connectivity over larger distances.

**Figure 2:**
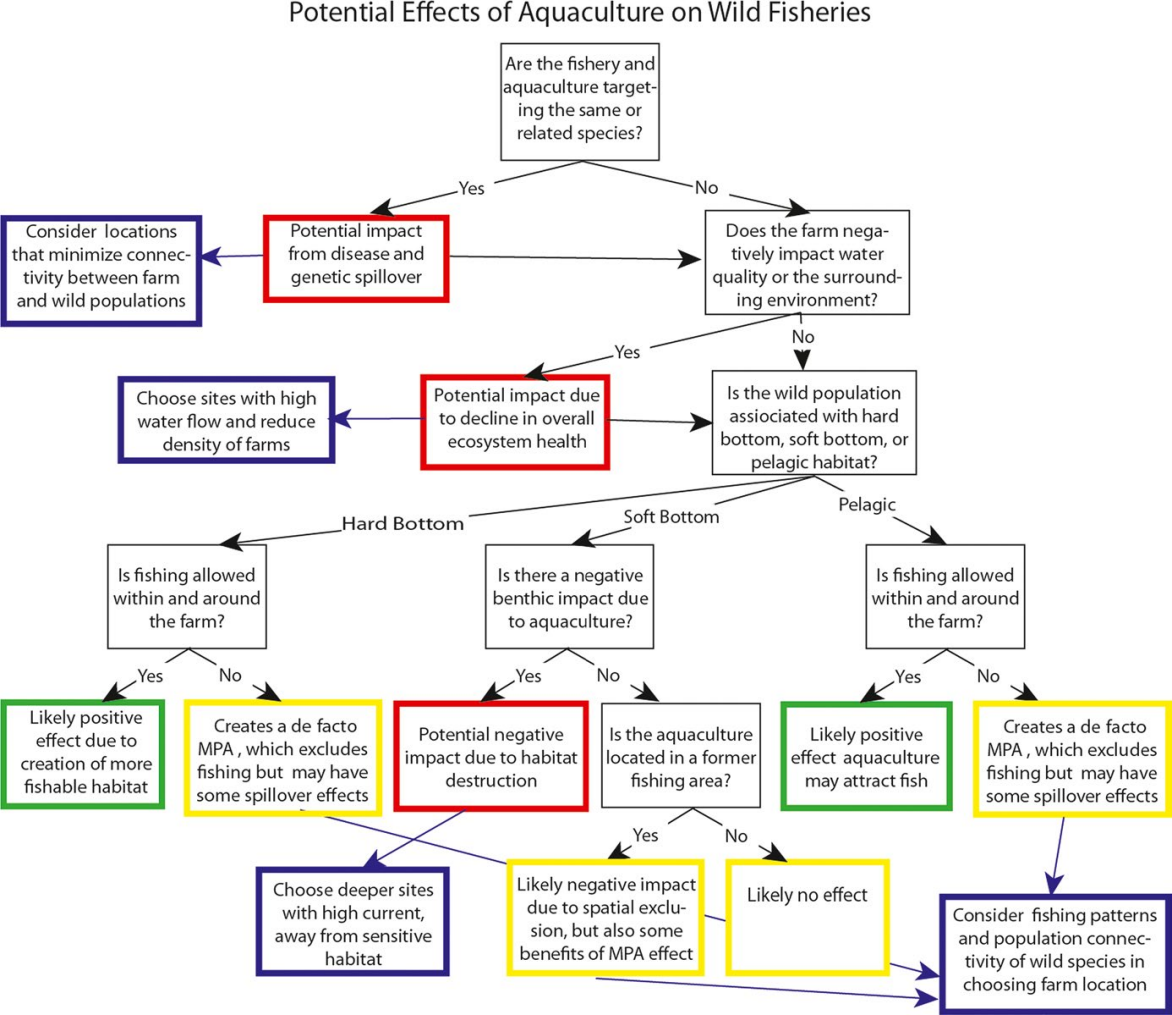
A flowchart for assessing the potential risks of an open‐ocean fish farm on wild fisheries, assuming best practice on‐farm management, and siting of the farm over soft‐bottom habitat. Black boxes represent questions about the attributes of the farm or environment that affect the outcomes; red, yellow, and green boxes represent potential (not mutually exclusive) effects on wild fisheries (indicating a risk of negative effects, neutral or mixed effects, and positive effects, respectively); and blue boxes represent potential spatial planning solutions to help mitigate risks. See text for supporting references

Aquaculture can have both positive and negative impacts on wild fisheries depending on farming methods, species, regulations, and environmental characteristics. Specifically, aquaculture can negatively impact the health of fish stocks by introducing disease and escapees that can interbreed with wild stocks (Hoagland, Jin, & Kite‐Powell, 2003; Tisdell, 2003); affecting food webs (Gibbs, 2004); and by degrading water quality and habitats via farm effluent and habitat conversion (Naylor et al., 2000). Avoiding aquaculture development in areas that are known to host high densities of target fish species can potentially reduce some of these risks. Furthermore, aquaculture can also potentially benefit wild fisheries by creating structure that could be utilized as habitat by target species or their prey, and by adding food and nutrients to the ecosystem, which could increase productivity or be consumed directly by target fish (Arechavala‐Lopez et al., 2011; Hehre & Meeuwig, 2016; Pitta et al., 2009). Several empirical studies in the Mediterranean (Bacher & Gordoa, 2016; Machias et al., 2006) have investigated the relationship between aquaculture and wild‐capture fisheries. Taken together, they have found either no impact or a positive effect. However, it is important to note that the Mediterranean is generally nutrient limited, so a modest influx of nutrients is more likely to boost productivity there than in more nutrient‐rich oceans. Figure 2 provides an example of how we can apply current knowledge to complex issues, like the effects of offshore aquaculture on fisheries, to evaluate potential risks and use spatial planning strategies to mitigate these risks and maximize positive synergies between objectives.

Siting decisions should vary based on the species being farmed, allowing for spatial plans that maximize potential benefits and minimize risks of aquaculture in any specific area. For example, placing kelp and bivalve farms in areas known to have high nutrient levels from other human sources could provide ideal growing conditions and benefit the surrounding environment. Conversely, finfish farms should likely be avoided in close proximity to particularly sensitive conservation areas, where any risk of pollution may be less acceptable. Further exploration of the ecological relationships between aquaculture, wild fisheries, and conservation would be particularly useful for improving spatial planning models.

## 3 Recommendations and Conclusions

**Figure 3:**
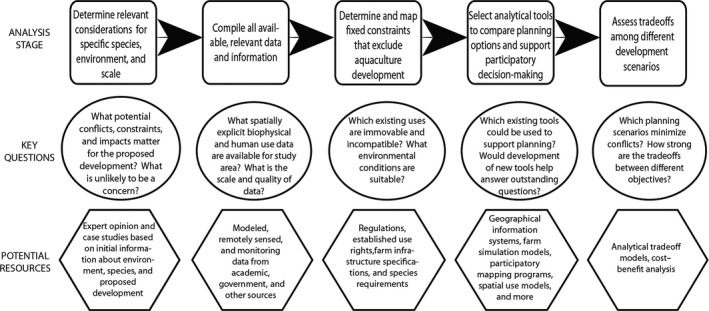
Recommended approach to incorporating scientific analysis to support spatial planning for development of offshore aquaculture. The rectangles contain key analysis stages; the circles and hexagons include important questions and potential resources, respectively, to help guide each of these stages

Offshore aquaculture is still an industry in its infancy, which makes it tempting to focus on information gaps and conclude that more research is necessary to understand its interactions with the surrounding environment. And while this is an area ripe with research opportunities, we can make informed siting decisions today about farm location and density. Furthermore, offshore aquaculture development is unlikely to wait for more research, making it essential that planning decisions leverage the best available information. Figure 3 provides guidance for organizing and distilling the most important ecological questions and analysis for aquaculture spatial planning. We highlight data and analytical tools that would inform a participatory planning process, acknowledging that this type of spatial analysis is only one part of a broader spatial planning process and that stakeholder engagement would be an essential component throughout.

As an initial step, it is important to narrow the focus to the most likely and relevant spatial planning issues for a specific development or region. Given specified environmental conditions, cultured species, and production goals, we can identify and assess when particular issues warrant further investigation, and when they are unlikely to be a concern For example, benthic deposition is unlikely to be a concern for a bivalve farm located in deep waters with high current, but should be more closely assessed for a finfish farm in relatively shallow or calm water. Table 1 provides a qualitative assessment of several key environmental risks, along with spatial planning strategies for reducing these risks, and available analytical tools if further evaluation is necessary. It is important to note that aquaculture technology is constantly improving, and new solutions are being introduced that mitigate environmental concerns. Therefore, planning that minimizes the environmental risks we encounter today will likely see even better performance in the future.

**Table 1:** Several key environmental risks for fed, unfed, and autotrophic aquaculture that can be mitigated by spatial planning, along with planning strategies that are likely to minimize risk, and examples of available analytical tools that can be used to evaluate these risks. We also qualitatively assess the overall risk of each environmental issue when aquaculture is well planned, that is, assuming that the listed risk reduction strategies are incorporated into spatial planning processes and that farm operations are well‐managed. See main text for supporting references

Environmental risk	Aquaculture types affected	Risk reduced by:	Overall risk for well‐planned offshore aquaculture	Available analytical tools
Benthic Impact	Fed, unfed	Choosing sites with high current and/or deeper waterAvoiding sensitive benthic habitats	Low	Aquaculture modeling software, such as Depomod, AquaModel, and the FARM model
Disease Outbreak	All	Reducing connectivity between farms growing similar speciesLocating farms away from habitat of native populationsReducing density of farms	Moderate	Oceanographic models, such as Regional Ocean Modeling Systems (ROMS); species distribution mapping
Water Column Pollution	Fed	Locating farms in environments with high natural productivity and low levels of existing nutrient pollutionUsing multitrophic farming techniquesReducing density of farms	Low	Aquaculture modeling software, such as Depomod and AquaModel
Marine Mammal Interactions	All	Locating farms away from marine mammal haul outs, migration routes, and important foraging grounds	Low risk of entanglement; moderate risk of behavioral change	Spatial analysis of wildlife movement patterns
Food and Nutrient Depletion in the Water Column	Unfed and autotrophic	Locating farms in areas with high natural productivityReducing density of farms	Low	Ecopath modeling

Data, analytical models, and planning tools can help guide development, but the final steps of spatial planning rely intrinsically on the values that people place on different outcomes. Using analyses such as tradeoff modeling can identify planning solutions that minimize conflict and also provide insight about the strength of unavoidable tradeoffs among objectives that cannot be resolved solely by efficient spatial planning (Lester et al., 2013). However, these analytical approaches can only provide guidance on the relative advantages of different development plans; managers and developers will ultimately have to make decisions about the type, location, and number of farms in a region based on societal risk tolerances and preferences across different objectives.

In general, we conclude that the profitability of an aquaculture farm and the potential environmental risks and impacts will vary substantially across regions and are influenced by the number and density of farms. In addition, the most important planning considerations depend on the species being farmed and the specific ecology and environmental conditions of the farm location. Since different species react in various, and often complementary ways to their surrounding environment, it is important to consider not just the total amount of aquaculture in an area, but also the diversity of farming methods and species. While grouping of similar farms together or the development of large monoculture farms may appear to be more valuable to the aquaculture industry due to efficiency gains and economies of scale, this tendency toward consolidation may increase environmental impact and disease risks. A large literature, primarily focused on terrestrial systems, has suggested that increased diversity can lower disease risk (e.g., Keesing, Holt, & Ostfeld, 2006) and reduce the need for chemical inputs in agroecosystems (e.g., Smith, Gross, & Robertson, 2008). Further, promoting the farming of diverse species not only has the potential to alleviate some environmental concerns, but also to create a more resilient industry (Troell et al., 2014), better placed to remain productive in our changing world.

## Conflict of Interest

None declared.
